# Measuring air quality in smoking and nonsmoking areas of Nevada casinos (Reno/Sparks): Potential exposure of minors to secondhand smoke

**DOI:** 10.5620/eaht.2024014

**Published:** 2024-04-16

**Authors:** Eric Crosbie, Sara Perez, Johnny Hartman, Lisa Sheretz, Neil E. Klepeis

**Affiliations:** 1 School of Public Health University of Nevada Reno, Reno, NV, United States; 2 Ozmen Institute for Global Studies, University of Nevada Reno, Reno, NV, United States; 3 Northern Nevada Public Health, Reno, NV, United States; 4 Education, Training, and Research Inc.(ETR), Scotts Valley, Sacramento, CA, United States; 5 San Diego State University Research Foundation (SDSURF), San Diego, CA, United States

**Keywords:** smoking, casinos, air quality, exposure, minors

## Abstract

To understand the potential exposure to tobacco smoke in Washoe County (Reno/Sparks), Nevada casinos by measuring air quality in smoking areas relative to non-smoking/non-gaming areas in which minors may be present. To act as a pilot study in community-based health research and policy campaigns by evaluating low-cost air monitors to measure personal secondhand smoke (SHS) exposure. We used customized mobile apps, AtmoTube PRO Air Monitors, and hand clickers to measure the timing and minute-by-minute levels of PM_2.5_ (a tobacco smoke marker). The app was used to record the number of smokers, minors, and total patrons associated with ~10-minute sequential time periods in standardized casino locations, including outdoor areas, slots, tables, restaurants, bars/lounges, arcades, among others. Between April and May 2022, we successfully visited 14 casinos and 18 distinct types of indoor casino locations. We found high PM_2.5_ peaks in casino locations even with zero, or a low percentage of, observed active smokers, including in both gaming/non-gaming areas. Indoor areas, regardless of smoking/non-smoking areas, consistently had higher PM_2.5_ levels than outdoor background levels. Indoor locations had median PM_2.5_ levels up to 18 times higher than the lowest outdoor background levels. Minors were present throughout all casino locations, and thus were likely exposed to elevated PM_2.5_ levels. Potential PM_2.5_ exposures due to smoking can be high regardless of ventilation systems. Small proportions of smokers in a location can lead to high levels of exposure. Establishing comprehensive smoke-free casinos is the only way to protect against SHS harms.

## Introduction

Secondhand tobacco smoke (SHS) exposure kills more than 400 infants and 41,000 adult nonsmokers per year in the U.S., while 58 million nonsmokers remain exposed to SHS [1]. Public establishments have attempted to introduce expensive and sophisticated ventilation systems for decades but systematic reviews along with notable government and non-governmental agencies and bodies have declared that there is no safe level of SHS [2, 3]. In response, governments around the world have enacted smoke-free policies in public places [4], establishing 100% smoke-free clean air in hospitals, hotels, government buildings, educational facilities, bars and more recently multi-unit housing units and outdoor places such as beaches and parks. [5, 6].

However, casinos are one of the remaining public places where smoking is allowed in which a large number of gaming establishments still permit indoor smoking [7]. Surprisingly, there is relatively little evidence published on casino air quality and exposure of minors, casino employees and other visitors to airborne pollutants in SHS such as PM_2.5_ (fine airborne particles) [8]. An extensive review of SHS in casinos concluded the exposure of employees and patrons to SHS in casinos poses a significant, preventable risk to health [7]. Other evidence shows PM_2.5_ levels can be high in casinos that allow smoking, [9-11] in which casino employees, patrons, and children are being exposed to hazardous levels of toxic substances present in SHS [12]. Even brief SHS exposures have been associated with adverse respiratory and cardiovascular health effects, [13] as minutes or hours of passive smoking exposure is nearly as harmful as active smoking [14].

While relatively few smoke-free casinos exist in the U.S. [15], several casinos and tribal casinos have expressed interest in becoming smoke-free in light of the adverse health effects associated with SHS [16]. As of February 2024, 20 states have passed laws requiring 100 % smoke-free gambling venues [15]. Meanwhile in Nevada, casinos are establishing smokefree gaming areas inside casinos (e.g. sportsbooks, poker rooms) and in September 2020, the Park MGM in Las Vegas went 100 % smoke-free [17]. Despite these encouraging voluntary efforts, the 2006 Nevada Clean Indoor Air Act (NCIAA), which prohibited smoking in most public places such as government buildings, schools, and restaurants, still permits smoking in casino gaming areas in Nevada [18]. Given that most casinos in Nevada advertise their properties as family friendly establishments, the NCIAA is increasingly seen as out of date. Families choose to frequent the restaurants, arcades, spas, bowling allies, among other family activities [19]. We are not aware of studies that have focused on the exposure to minors in casinos or the application of low-cost real-time mobile sensors, and accompanying mobile apps, which have the potential to support widespread community deployment and data-driven community activity. Thus, this study aims to (1) contribute new data on the exposure of minors to SHS in the northern Nevada area of Reno and Sparks, and (2) evaluate communityaccessible exposure assessment methods. In fulfilling these aims, we seek to inform the public, casino employees and policymakers about the harms of SHS in casinos, especially for minors who typically do not have the choice to avoid being harmed by SHS, and to provide pilot data for future community-based research and policy initiatives, including Citizen Science programs.

## Methods

### Approach

We sought to investigate the potential exposure of minors (under 18 years old) to secondhand smoke in Reno/Sparks casinos. Our specific research questions were: (1) How well can low-cost air monitoring devices be used in the field measurement of personal exposure to secondhand smoke?; (2) How high are PM_2.5_ concentrations in casinos relative to clean outdoor air?; (3) What is the association between PM_2.5_ levels and smoking activity; (4) Are minors present in areas where smoking is allowed or occurs?; (5) What are PM_2.5_ levels in smoking versus non-smoking and gaming versus nongaming areas of the casinos?; and (6) What PM_2.5_ concentrations could minors be potentially exposed to? We investigated these questions using field study procedures involving personal PM_2.5_ measurement and activity logging as described below.

### Field procedures

Between April and May 2022, we mounted a field campaign to measure PM_2.5_ levels in 15 targeted gaming and non-gaming establishments in Reno and Sparks, Nevada. We planned to visit the casinos as a normal patron would - walking around and spending time in different gaming and non-gaming areas. We also attempted to visit casino locations on the weekends, in which we expected higher activity and an increased presence of minors. Of these 15 establishments, 13 have both gaming and non-gaming locations within their establishments and two were completely non-gaming. The 13 establishments with gaming represent the total population of casinos in Reno and Sparks that have a minimum size of 30,000 square feet. These establishments and the planned visits on the weekends represent the most popular and highly visited gaming locations and time periods in the Reno and Sparks area.

We used the general methods previously developed and implemented by Klepeis et al. (2012; 2016) [11, 20] and Jiang et al. [9] to characterize the exposure of casino patrons and staff to PM_2.5_ concentrations. Generally, the monitoring procedure consists of carrying monitors in sequential ~10-minute visits to standard locations in the casino, starting and ending with outdoor locations to provide control (background) levels. Inside each establishment, up to six different locations were visited, including gaming/non-gaming and smoking/non-smoking locations. The outdoor areas were nearby the casino, such as sidewalks, balconies, driveways, etc. Indoor areas included at least one gaming area (e.g. tables or slots) with smoking as well as attempting to include at least one area without gaming and one non-smoking area.

For data-gathering instruments, we used a combination of a low-cost battery-powered portable sensor, two mobile apps, and hand clickers. We used the handheld clickers to count the number of smokers, minors and total patrons present at each location. To gather continuous 1-minute levels of PM_2.5_ during each casino visit, we used the AtmoTube PRO real-time air monitor (AtmoTech, Inc. San Francisco, CA, USA) which comes with an accompanying mobile app that we used to configure the monitor and sync 1-minute readings over Bluetooth. The AtmoTube PRO is quiet, light, and unobtrusive with a battery life that is sufficient for up to 12 hours of continuous 1-minute readings. We carried the AtmoTube PRO continuously throughout each casino visit to measure personal PM_2.5_ exposure and store the data on mobile phones equipped with the AtmoTube app. AtmoTube data were also automatically transmitted to a manufacturer-supplied cloud database.

The AtmoTube PRO particle sensor has been found to measure PM_2.5_ levels with high precision and 84 % to 95 % accuracy [21, 22]. Since readings can be affected by the type of particle source, we evaluated absolute PM_2.5_ measurement of the AtmoTube PRO specifically for SHS with a side-by-side laboratory comparison against a reference DustTrak instrument (TSI, Shoreview, MN) in which a cigarette was smoked in a small chamber. Whereas factory-calibrated DustTrak and SidePak monitors have been found to overestimate SHS PM_2.5_ levels by 3.3 to 4 times when compared to “gold standard” gravimetric measurements [9, 23], we found that the AtmoTube PRO *underestimated* the factory-calibrated DustTrak by about 3 1/3. Thus, we expect that unadjusted AtmoTube PRO readings in the field may only overestimate true PM_2.5_ concentrations for SHS by up to 20 %, which is in the range of acceptable measurement error. It should be noted that studies using uncalibrated DustTrak instruments or Sidepacks [8] are expected to overestimate SHS PM_2.5_ particle concentrations by 3 1/3 to 4 times.

To gather data on the timing of each visit to a casino location and the number of observed patrons made using handheld clickers, we used a custom mobile app called “Casino Logger” that was developed by Neil Klepeis for both iOS and Android smartphones using the AppSheet software and mobile app hosting service (Google LLC, Mt View, CA). We installed the Casino Logger app on mobile phones and used it to fill in forms with real-time field data during casino visits on the designation of a location as smoking or non-smoking, the precise time locations were visited, and the number of smokers, minors, and total patrons that were present in each location. Data from the Casino Logger app were automatically transmitted to an online Google Sheet database for later retrieval.

We used the following seven-step procedure to gather data during each casino visit: 1) Set up the AtmoTube PRO air monitor and mobile app prior to visiting the casino to gather readings every minute, 2) Attach the AtmoTube PRO monitor to clothing, backpacks, straps, or otherwise “wear” the monitor, 3) Prepare the Casino Logger App by entering casino name, AtmoTube PRO device ID, and date, 4) Make initial outdoor PM_2.5_ readings for ~10-minutes to obtain background (ambient) levels by visiting a nearby sidewalk or patio with no cars, smokers, or other sources [of air pollution?], 5) Enter and walk around inside the establishment and use the app to record counts of patrons for ~10-minute sequential periods in prescribed smoking and nonsmoking areas, and gaming and non-gaming areas, 6) Make final outdoor PM_2.5_ readings for ~10-minutes in the same outdoor location as before, and 7) Export the AtmoTube data as a CSV file for data analysis as a redundant backup to cloud-based storage.

We attempted to visit up to six distinct types of locations per casino and a mix of smoking/nonsmoking and gaming/non-gaming areas. Given that casinos had different sizes, layouts and types of rooms, we planned to visit at least one smoking and nonsmoking area and one gaming and non-gaming area per casino and with possible overlap between smoking/gaming activities.

### Data analysis

We used the R system and functions from the tidyverse R packages to create an aggregated database in a standardized format from all gathered data and analyze the air quality and activity logging data [24, 25], producing boxplot charts of PM_2.5_ concentrations and tables containing descriptive statistics of activity logging data. Boxplots describe the distribution of a continuous variable in which the “box” represents the inter-quartile range, the line inside the box is the median (50th percentile), the “whiskers” (line extending from the box) represent the range of values excluding extreme values, and the dots represent extreme (e.g., maximum) values. Boxplots are useful for generating a compact comparison of variable distributions, including the central tendency, spread, and extreme values, as a function of a categorical variable.

## Results

### Overview and protocol evaluation

With only a single case of data loss for one casino, we gathered data from 14 out of the original 15 targeted casinos in the Reno/Sparks areas. We successfully implemented the procedure described above for gathering 1-minute PM_2.5_ readings in indoor and outdoor casino areas with concurrent location and person-count data recorded on the “Casino Logger” app. Time-stamped logs precisely marked entry and departure times for each casino area for later analysis of areaspecific PM_2.5_. Data were successfully uploaded to the cloud, retrieved from smart phones, and used to create statistical summaries across casinos and locations visited. Supplementary Figure 1 illustrates field data from one of our casino visits, consisting of real-time personal PM_2.5_ levels and tagged time periods indicating the location of personal monitoring. As presented in Tables 1, we visited between 2 and 10 locations per casino and 18 distinct types of indoor casino locations, reflecting a broad range of locations that casino patrons typically visit. The total time spent in each type of indoor location ranged from 8 to 110 minutes. The number of smoking locations visited in each casino ranged from 0 to 5 with up to 269 minors observed in all locations of a certain casino and between 110 and 1096 total observed patrons per casino. For casinos that allow smoking, we observed 1.4 % to 20 % of patrons smoking in the locations we visited. For locations with observed smokers, per-location smoker proportions were 2 % to 15 %. Per-casino median PM_2.5_ levels ranged from 3 to 37 µg/m^3^ and maximum levels from 5 to 91 µg/m^3^, whereas per-location median and maximum levels were 1 to 56 µg/m^3^ and 3 to 91 µg/m^3^, respectively.

### Relative PM_2.5_ concentrations: outdoors, gaming, non-gaming areas

**Figure 1.**
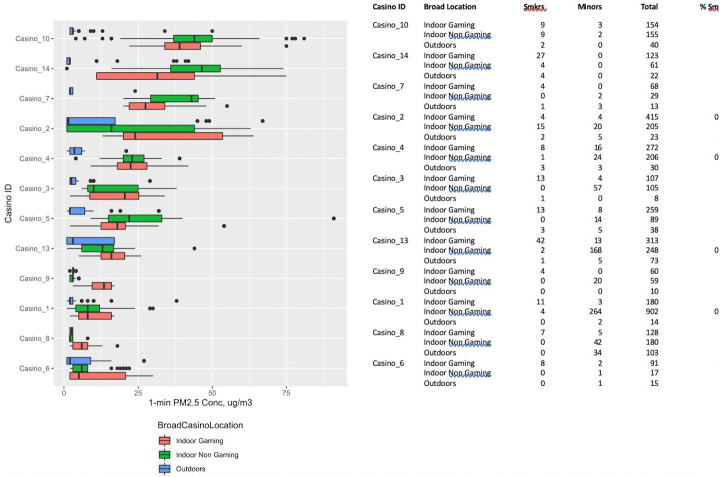
PM_2.5_ distribution (in μg/m^3^) and total people counts by broad casino location category

The per-location PM_2.5_ levels shown in Supplementary Table 1 indicate a clear elevation of particle levels when going from outdoor to indoor locations and from certain non-gaming locations to gaming locations. For example, median levels when outside, in a vehicle, in a bowling alley, ball room, and a play area were 1 to 3 µg/m^3^. In comparison, median levels at gaming tables, slots, and bar/restaurant locations were 20 to 57 µg/ m^3^. These differences are an apparent relative increase in particle level of about 7 to 60 times. Figure 1 presents distributions and statistics for PM_2.5_ levels and activity logs, respectively, aggregated into Indoor Gaming, Indoor Non-Gaming, and Outdoor locations. The boxplots are sorted from highest to lowest median concentration measured in Indoor Gaming locations. Regardless of casino, and the number of observed people and smokers, indoor levels were consistently higher than outdoors considering both central tendency and spread of the distributions. For some casinos with the highest indoor levels, the relative difference was up to about 50 times or higher. For nine casinos across the range of medians, nearly all indoor levels were higher than measured outdoor levels. While the effects of smoking bans were evident – with casino non-smoking areas having the lowest median indoor gaming and non-gaming levels – casinos with smoking fell across a broad range of median indoor levels. Some outdoor locations had occasional high levels (indicated by dots on the boxplots).

### Relative PM_2.5_ Concentrations: Smoking and Nonsmoking Areas

**Figure 2.**
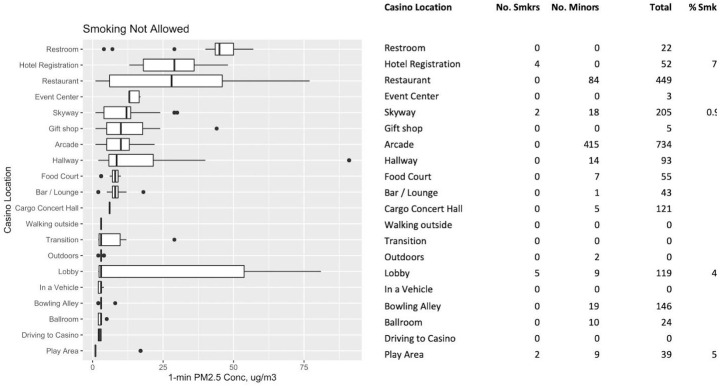
PM_2.5_ concentration distributions (in μg/m3) and total counts of people for locations in which smoking IS NOT allowed.

**Figure 3.**
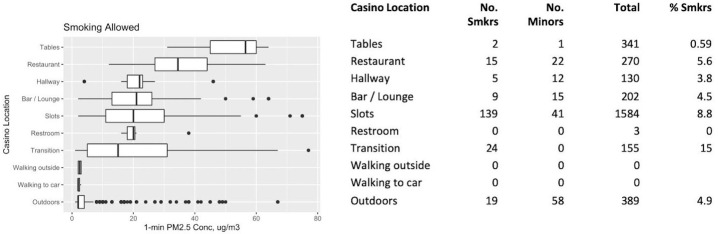
PM_2.5_ concentration distributions (in μg/m3) and total counts of people for locations in which smoking IS allowed.

Figures 2 and 3 present distributions of PM_2.5_ levels and statistics from people-count data for locations where smoking was not allowed and allowed, respectively. The boxplots are sorted top to bottom for locations having the highest to lowest median PM_2.5_ concentration. The smoking policy for each location was recorded based on observation of signs and casino-provided materials. Some non-smoking locations still had some of the largest observed PM_2.5_ concentrations that were comparable to the highest smoking-allowed locations (e.g., Tables), including Restroom, Hotel Registration, and Restaurant (median levels above 25 µg/m^3^). In most non-smoking areas, no smokers were observed, even though PM_2.5_ levels could be high (e.g., Restroom, Restaurant). In some of the non-smoking locations, such as in Hotel Registration, smokers were still observed despite the apparent no-smoking policy. Other designated non-smoking areas in which we observed smokers were the Skyway, the Lobby, and Play Area. These locations all had peak levels well above outdoors. Hallway, Bar/Lounge, and Slots were smoking-allowed areas with observed smokers and with comparable ranges of elevated PM_2.5_ concentrations (medians of 20-25 µg/m^3^), which were generally lower than the smoking Restaurant and Table areas (medians over 35 µg/m^3^). In smoking-allowed areas, we observed as many as 8 % to 15 % of patrons smoking (Slots area and Transition between locations, respectively) although high levels (e.g. Tables, two observed smokers) did not correlate with higher numbers of smokers. Outdoor locations that allowed smoking and had observed smokers had low median levels, but a number of very elevated peak levels as large as concentrations observed in the smoking Restaurant and Table areas.

### Presence and Potential Exposure of Minors

One of the main aims of this study is to characterize potential exposure of SHS to minors in Reno/Sparks casinos. The key measure for this evaluation is the observed presence of minors in locations in which there were elevated PM_2.5_ concentrations relative to clean background levels, such as those observed outdoors with no proximate smokers. A higher number of observed minors in PM_2.5_-elevated areas translates to higher potential population exposure. Our data show that large numbers of minors are visiting Reno/Spark casinos (Tables 1). Two casinos had hundreds of minors present and six had 20 or more minors at the times of our site visits. The most minors were observed in Arcade and Restaurant locations (415 and 106, respectively). Between 14 and 41 minors were observed in Bar/Lounge, Bowling Alley, Hallway, Skyway, and Slots areas. As illustrated in Figure 1, minors could be present in both gaming and non-gaming areas, in smoking and nonsmoking areas, and with smokers present or not present and still could be in contact with median PM_2.5_ levels substantially above much lower background outdoor levels by as much as 10 to 40 µg/m^3^.

## Discussion

Our examination of air quality in casinos in the northern Nevada area of Reno and Sparks revealed high levels of particulate matter from smoking in most casino locations compared to outdoor levels, including in both smoking and nonsmoking areas. While these findings are consistent with other studies [26-29], this study provided a breakdown of various casino locations (arcades, restaurants) illustrating the exposure of SHS in several locations, most notably family friendly places where minors are present. We found statistically significant differences between casinos using one-way ANOVA but there is a lot of unexplained variation (due to casino differences, source proximity, flows, etc.). However, PM_2.5_ levels were elevated regardless of casino characteristics.

This study also deliberately counted the number of smokers and minors present in each casino location. Our study showed that minors were found to be present throughout all casino locations, regardless of smoking and non-smoking areas, and thus, are exposed to SHS, especially those with acute health risks. We observed relatively small portions of active smokers (1.4 % to 20 %) in casinos that allowed smoking, which is also similar to active-smoker proportions reported elsewhere [9, 11, 20]. Consistent with these findings, a small proportion of active smokers can still contribute to high levels of PM_2.5_ and contaminate non-smoking areas [20]. Unseparated non-smoking areas are still contaminated by SHS, pointing to the inadequacy of ventilation systems and partial smoking bans in casinos to protect nonsmoking-patrons and employees.

This is the first known study to use the AtmoTube, an off-the-shelf, low-cost, user-friendly consumer device, and custom mobile apps to measure real-time personal exposure and exposure contexts in casinos. Previously used expensive and noisy monitors that are difficult to operate and retrieve data from have likely inhibited the use of community-based personal exposure monitoring. We expect that our successful approach using low-cost and accessible sensors could be implemented widely to enable members of the public to characterize personal SHS exposure, easily share their data, and contribute to publicly available knowledge that can be presented directly to policy makers and inform new indoor air quality policies or enforcement of existing policies.

### Policy implications

Babb et al. (2015) reported the dramatic effects of comprehensive smoke-free policies on air quality in casinos, seeing quick and significant reductions of PM_2.5_ concentrations by as much as 80 to 90 % following a few months of implementation [7]. Klepeis et al. (2016) reported a striking reduction of 98 % in PM_2.5_ exposure in smoking areas following implementation of a complete smoking ban in the Win-River Resort and Casino where PM_2.5_ levels fell from 60 to 87 µ g/m^3^ to only 1-2 µ g/m^3^ [20].

Studies have highlighted the gaps in protection offered by the 2006 NCIAA, as partial smoking bans in casinos are ultimately ineffective to protect employees and patrons from SHS harms [7, 10, 27, 28]. Given that several casinos in the Reno and Sparks area advertise their establishments as family-friendly, they should be aware that even during brief visits by minors in smoking and non-smoking areas as well as in both gaming and non-gaming areas may be exposed to harmful PM_2.5_ levels, regardless if active smokers are present. These findings can also be useful for casino employees to understand the health and occupational risks of SHS exposure in casinos. Casino owners should also be aware that casino employees exposed to SHS leads to more days off work and loss of productivity [30].

Given the gaps in protection offered by the 2006 NCIAA, it is important for policymakers in Nevada to amend the NCIAA and prohibit smoking in all public places including casinos and bars in Nevada. Policymakers in Nevada have stated they would strengthen the NCIAA if their constituents supported such a move [31] and a recent survey of local Reno and Sparks citizens found that 74 % of non-smokers indicated they would favor a law prohibiting smoking in all casinos [19]. If policymakers are unable to amend the NCIAA and support for smoke-free casinos continues to increase, health advocates could also help create and support a statewide ballot measure to bring this decision to Nevada voters.

### Limitations

The personal exposure data collected in this study are not intended to provide high accuracy in absolute measures of particle exposure due to SHS. They are useful to evaluate broad and relative increases in particle exposure that can occur in casinos due to smoking behavior, using estimates of the accepted PM_2.5_ indicator for tobacco smoke exposure, and to identify community needs for exposure mitigation. Exposure depends on time spent in locations with elevated concentrations of PM_2.5_. In this study, we used indirect measures of particulate exposure based on PM_2.5_ levels and observations of the presence of minors. We did not explicitly measure the personal exposure of minors. We concluded that there was the potential for sizeable exposure in terms of magnitude and numbers of minors present but did not quantify the precise personal exposure or population exposure. We also did not evaluate the ventilation systems in these establishments, which could affect exposure to PM_2.5_ levels. However, there is no known safe level of tobacco smoke regardless of the ventilation system.

## Conclusions

Particulate matter in casinos with smoking can be high in any casino regardless of types of ventilation and air filtering equipment that may vary between casinos. These levels can be high even when few or no active smokers are present in a given location. Minors can be exposed to elevated particulate matter concentrations from smoking in designated non-gaming or non-smoking areas of casinos. Establishing comprehensive smoke-free casinos is a recommended way to protect against SHS harms [7].

## Figures and Tables

**Table 1.** Descriptive statics for each casino visited.

SiteID	No. of locations	No. of smoking-allowed locations	Total No. of smokers	Total No. of minors	No. of total people	% Smokers	Median PM_2.5_ Conc., μg/m^3^	Max PM_2.5_ Conc., μg/m^3^
Casino_1	6	2	15	269	1096	1.4	6	38
Casino_2	5	4	21	29	643	3.3	20.5	67
Casino_3	5	2	14	61	220	6.4	8	38
Casino_4	5	5	12	43	508	2.4	22	42
Casino_5	4	3	16	27	386	4.1	12	91
Casino_6	5	3	8	4	123	6.5	5	30
Casino_7	8	5	5	5	110	4.5	22	55
Casino_8	8	4	7	81	411	1.7	3	77
Casino_9	8	3	4	20	129	3.1	3	18
Casino_10	10	5	20	5	349	5.7	37	81
Casino_11	4	0	0	19	172	0	4	5
Casino_12	2	0	0	7	169	0	6	6
Casino_13	8	4	55	186	746	7.4	13	44
Casino_14	6	5	49	0	249	20	24	75

## Supplementary Material

The data underlying this article are available in the article and in its online supplementary material.
