# Supplementary material for: Measuring air quality in smoking and nonsmoking areas of Nevada casinos (Reno/Sparks): Potential exposure of minors to secondhand smoke
Source: Environ Anal Health Toxicol. 2024 Apr 16;39(2):e2024014. doi: 10.5620/eaht.2024014 (PMC11294658; doi:10.5620/eaht.2024014)
Supplement: Supplementary file 1 [file eaht-39-2-e2024014-Supplementary-Table-1.pdf]

Supplementary Material

Supplementary Table 1. Descriptive statistics for each casino location visited.

Location	Total Minutes Spent	No. Smoking -Allowed	Total No. of Smokers	Total No. of Minors	Total No. of People	% Smokers	Median $PM_{2.5}$ Conc., μg/m^3	Max $PM_{2.5}$ Conc., μg/m^3
Arcade	110	0	0	415	734	0	10	22
Ballroom	13	0	0	10	24	0	3	5
Bar / Lounge	94	6	9	16	245	3.7	19.5	64
Bowling Alley	10	0	0	19	146	0	3	8
Bundox Bocce	9.9	0	0	14	127	0		
Cargo Concert Hall	10	0	0	5	121	0	6	6
Driving to Casino	6.3	0	0	0	0		2.5	3
Event Center	18	0	0	0	3	0	13	17
Food Court	13	0	0	7	55	0	8	10
Gift shop	13	0	0	0	5	0	10	44
Hallway	39	2	5	26	223	2.2	19	91
Hotel Registration	11	0	4	0	52	7.7	29	48
In a Vehicle	32	0	0	0	0		3	4
Lobby	47	0	5	9	119	4.2	3	81
Outdoors	400	32	19	60	389	4.9	3	67
Play Area	17	0	2	9	39	5.1	1	17
Restaurant	210	3	15	106	719	2.1	31	77
Restroom	34	1	0	0	25	0	43.5	57
Skyway	20	0	2	18	205	0.98	12	30
Slots	190	15	139	41	1584	8.8	20	75
Tables	8.2	1	2	1	341	0.59	56.5	64
Transition	55	20	24	0	155	15	13	77
Walking outside	4.8	2	0	0	0		3	3
Walking to car	10	1	0	0	0		2	3

Copyright © 2024 The Korean Society of Environmental Health and Toxicology/Korea Society for Environmental Analysis This is an Open Access article distributed under the terms of the Creative Commons Attribution Non-Commercial License (https://creativecommons.org/ licenses/by-nc/4.0/) which permits unrestricted non-commercial use, distribution, and reproduction in any medium, provided the original work is properly cited.
