# Supplementary material for: Measuring air quality in smoking and nonsmoking areas of Nevada casinos (Reno/Sparks): Potential exposure of minors to secondhand smoke
Source: Environ Anal Health Toxicol. 2024 Apr 16;39(2):e2024014. doi: 10.5620/eaht.2024014 (PMC11294658; doi:10.5620/eaht.2024014)
Supplement: Supplementary file 2 [file eaht-39-2-e2024014-Supplementary-Figure-1.pdf]

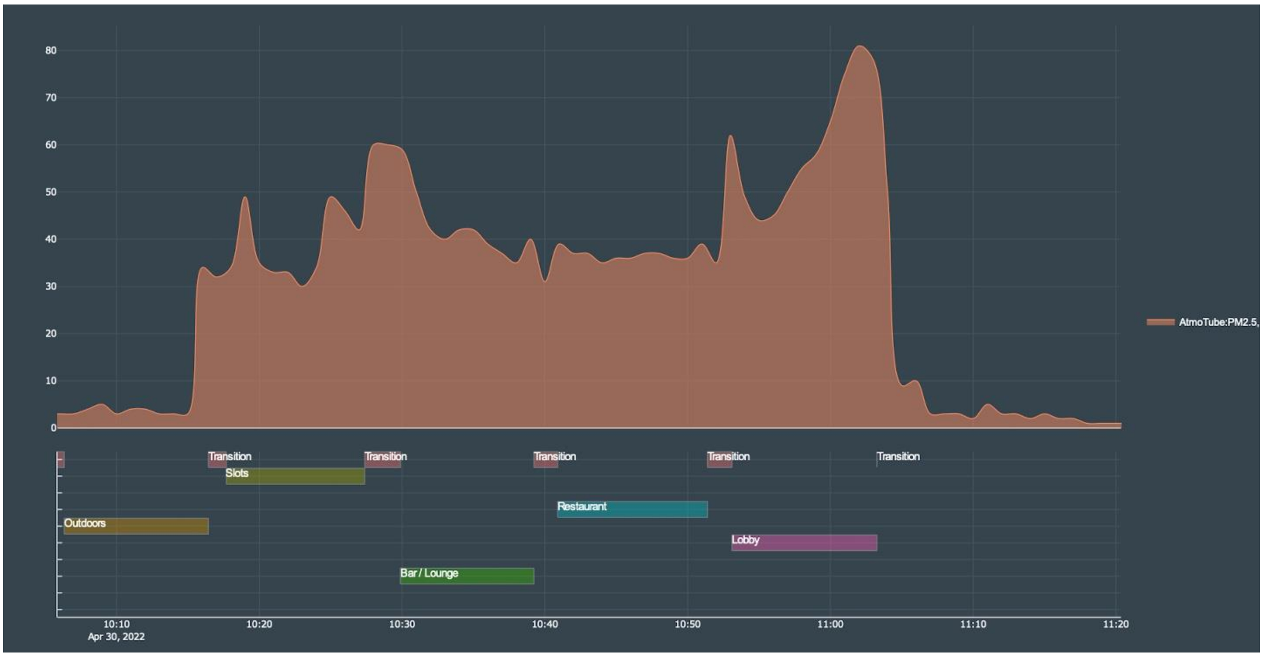

Supplementary Figure 1. Example time series of personal $PM_{2.5}$ concentrations in μg/m^3 measured by an AtmoTube PRO air monitor during a casino visit (spline fit 1-minute readings). Locations visited are shown as bars below the time series. The locations and other observational data were recorded in real-time using a custom "Casino Logger" mobile app.
